# Indigestion

**Published:** 1895-12

**Authors:** 


*Indigestion.* By GEORGE HERSCHELL, M.D. Second Edition. Pp. viii., 343. London: BAILLIÈRE, TINDALL AND COX. 1895.

It was only to be expected that a second edition of this book should have been quickly demanded. It gives a concise account of the modern methods of study and treatment of the disorders of the digestive apparatus, and is likely to be of much service to the busy practitioner in dealing with this frequent and troublesome group of conditions.

We are gratified to find that in the treatment of ulcer of the stomach the author trusts, during the first stage, entirely to the use of nutrient enemata; but we are astonished to note that this stage lasts "from four days to a week" only, whereas we should expect that it might very advantageously be lengthened to "as long as the patient can be induced to submit." We have searched, but in vain, for an analysis of the results of modern methods of the treatment of ulcer: these must show a great improvement on the figures given in the older text-books before the habitual use of nutrient enemata and the process of peptonisation became the rule.

